# Prevalence and Associated Factors of Burnout syndrome among Nurses in Public Hospitals, Southwest Ethiopia

**DOI:** 10.4314/ejhs.v31i3.11

**Published:** 2021-05

**Authors:** Alemayehu Sayih Belay, Melak Menberu Guangul, Wondwossen Niguse Asmare, Sitotaw Kerie Bogale, Gizachew Ayele Manaye

**Affiliations:** 1 Department of Nursing, College of health sciences, Mizan-Tepi University, Mizan-Teferi, Ethiopia; 2 Department of Psychiatry, College of medicine and health sciences, Bahir Dar University, Bahir Dar, Ethiopia; 3 Department of Adult health nursing, College of health sciences, Bahir Dar University, Bahir Dar, Ethiopia; 4 Department of Medical laboratory, College of health sciences, Mizan-Tepi University, Mizan-Teferi, Ethiopia

**Keywords:** Burnout Syndrome, Factors, Nurses, Prevalence, Ethiopia

## Abstract

**Background:**

The Burnout syndrome has been defined as a response to the chronic work-related stress typically found in professionals working in care service organizations. Therefore, this study aimed at assessing the prevalence of burnout syndrome and factors associated with burnout syndrome among nurses in public hospitals, Southwest Ethiopia, 2018.

**Method:**

An institutional-based cross-sectional study was conducted from February 1^st^, 2018 to April 1^st^, 2018. Total number of nurses who fulfill the inclusion criterias were enrolled. Data was collected using the predesigned tool. Data were entered using EPI INFO version 7 and was exported to statistical packages for social science (SPSS) version 21.0 for analysis. Logistic regression was employed and odds ratio with a 95% confidence interval was used. Variables with a P value of less than 0.05 was considered statistically significant.

**Results:**

A total of 282 eligible nurses were enrolled in the study with a mean age of 28.71 [SD ±7.047]. The prevalence of burnout syndrome among nurses was 96(34%). Predictor variables like; educational status, job title, work experience, fatigue, and social support were found to be strong predictor variables for burnout syndrome.

**Conclusion:**

This study revealed that a considerable proportion of nurses had burnout syndrome. Therefore, improved educational status and strong social support should be encouraged among nurses working in the health setting for the betterment of health care services, job satisfaction and quality of care in general.

## Introduction

The burnout syndrome is a psychological syndrome emerging as a prolonged response to chronic exposure to work-related stressors. Burnout syndrome first appeared around the early 1970s, aimed at determining the process of physical and mental deterioration in professionals working in areas such as health care, teaching, emergency legal services (1).

According to Maslach and Jackson, burnout syndrome is a process of the three domains of sub-syndromes, which can be characterized by low personal accomplishment, high emotional exhaustion and high depersonalization (2). The first domain is emotional exhaustion (EE), which refers to a situation of exhaustion, feeling of being strained and inability to be empathetic to others. The second domain is cynicism (CY) or depersonalization, which can be defined as the development of negative feelings and of cynical attitudes and feelings towards their patients and addressees of their work. The third domain is low personal accomplishment (PA), which refer to the trend towards provider evaluates his/her work negatively irrespective of patient outcomes. Such an evaluation especially affects their ability to do their work and the relationship with the people they are attending to (3).

World Health Organization (WHO), classifies burnout syndrome as an “occupational phenomenon” in International Classification of Diseases 11^th^ revision (ICD-11) (4). It is a global concern and work-related stress commonly experienced in health care professionals that occurs mainly among nurses whose work involves constant demands and intense interactions with people who have physical and emotional needs (5). The overall prevalence of burnout syndrome among global nurses was 11.23%. Sub-Saharan African region, Southeast Asia and Pacific had the highest rate (13.68%) followed by Latin America and the Caribbean (10.51%) of burnout syndromes while Europe and Central Asia region had the lowest (6). Another study also revealed that about 34.6% of nurses develop burnout syndrome (7).

Several factors have been found to be associated with the development of burnout among nurses, such as work experience, age of the nurse, and hierarchy of nurse (8). Others are excess workload at health institutions and household (9), emotional stress, unevaluated work and underpayment (10), conflicts with staff, lack of social support (9, 11) conflict with other nurses and conflict with physicians (11).

Several studies have revealed that African HCWs have low job satisfaction (12), which increases the risk of burnout and this in turns contributes for different negative consequences. For instance, it influence the nurses to change the working environment frequently and even to go away from their current profession (13). The most common negative consequences of burnout are particularly the deterioration of performance and productivity, decreasing quality of health services, health problems, frequent absence, accidents at work, and use of alcohol and medicines (14).

In Ethiopia, as some of the studies revealed that the prevalence of burnout syndrome were varied across health institutions in different geographical areas, where 13.7% (15), 50.4% (16) and 36.7 % (17) in Gondar University Hospital, public hospitals of Amhara region and Jimma University Teaching Hospital respectively. Even though few studies on prevalence of burnout syndrome in Ethiopia have been previously done (15–18) still it is limited specifically at southwest Ethiopia. Therefore, this study aimed to assess the prevalence and associated factors among nurses in public hospitals, southwest Ethiopia.

## Materials and Methods

**Study design and period:** An institutional based cross-sectional study design was carried out at public Hospitals from February 1^st^, 2018 to April 1^st^, 2018.

**Study setting:** The study was conducted in public hospitals located in three zones of Southwest Ethiopia including Mizan-Tepi University Teaching Hospital, Gebretsadik Shawo General Hospital and Tepi GeneralHospital. All of these hospitals have immunization department, delivery ward, inpatients wards, outpatient departments, antenatal and post-natal clinic. Currently, Mizan-Tepi University Teaching Hospital is expected to provide service for more than 829,000 populations. While Gebretsadik Shawo General Hospital and Tepi General Hospitals are expected to provide care for more than 500,000 populations. Mizan-Tepi University Teaching Hospital, Tepi General Hospital and Gebretsadik Shawo General Hospital has 106, 94 and 82 nurses respectively whose work experience is one or more years.

**Study Participants:** To be included, participants had to fulfil the following criteria: (1) have a minimum of year work experience in the same workplace or a similar health setting, (2) age 18 years and older and, (3) being nurse and working in only clinical departments of public hospitals. No participants were critically ill and unable to give response during data collection time and all of them were included.

**Data collection tools and procedure:** The data was collected using self- administered questionnaire by six trained health professionals' and three supervisors. The questionnaire was pretested among 5% of the sample size in health centers which were out of the study settings. The coherence and skipping pattern of the questionnaire was corrected after the pretest.

To assess levels of burnout, the English version of Maslach's Burnout Inventory-Human Services Survey (MBI-HSS) was used, which comprises 22 items regrouped into 3 subscales: emotional exhaustion (EE; nine items), depersonalization (DP; five items), and personal accomplishment (PA; eight items). Each item will be answered on a 7-point Likert scale ranging from “never” (= 0) to “daily” (= 6). The results of the inventory consist of three separate scores, one for each factor or subscales. The total scores of each sub-scale (emotional exhaustion, depersonalization, and personal accomplishment) were summed up first and then those scores ≥27, 17–26, and 0–16 in EE ≥13, 7–12, and 0–6 in DP, and 0–21, 22–38, and ≥39 in personal accomplishment categorized as high, moderate, and low respectively. A combination of cutoff values of high scores ≥27 for EE, ≥10 for DP, and ≤33 for PA, was considered to correspond to a high level of burnout. The MBIHSS is a self- administered questionnaire, has been reliable and valid (19, 20).

To assess social support, Oslo Social Support Scale was used. Oslo Social Support Scale Score is ranged from 3–14 with a score of 3–8 = poor social support; 9–11 = intermediate social support; and 12–14 = strong social support (21).

For psychological distress (anxiety, depression and somatization), Self-Reporting Questionnaire version 20 (SRQ 20) was used, which was developed by World Health Organization (WHO) for low and middle-income countries including Ethiopia (22). It has a “YES” or “No” questions and can be used as self-administered or interviewer administered questionnaire (23). SRQ 20 is a 20 item questionnaire commonly used to screen anxiety, depression and somatization symptoms (24). We used the cut-off point 8 based on the finding from the validation study of SRQ-20 which gave the highest sensitivity and specificity (23).

Sleep disturbance was measured by Athens insomnia scale (AIS). The AIS is 8-item self-reported questionnaire that indicates insomnia within the past month and scores each ranging from 0 to 3 (0 score equals better and 3 is worst). The total score greater than or equals to 6 indicates insomnia (25).

**Data analysis:** The data was entered using EPI INFO version 7 and was exported to statistical packages for social science (SPSS) version 21.0 for data cleaning and analysis. Bivariate logistic regression was done to see the degree of association between independent variables (socio-demographic, organizational related factors, psycho-social related factors) and dependent variable (burnout syndrome) were assessed. Variables with P value of <0.05 was recruited for multivariate analysis to control the effects of confounding. Finally, the results considering confidence level of 95% and P value of <0.05 were taken as a strong predictor for burnout syndrome. The results were presented in the form of tables, figures, and summary statistics.

**Ethics approval:** The study was approved by the research standing committee of Mizan Tepi University with a reference number CHS/0246/15/19. Permission letter was taken from each hospital administration office. After explaining the objectives of the study, written consent was obtained from each study participant. Data were collected with strict privacy and assuring confidentiality.

## Results

**Socio-Demographic Characteristics of the Respondents:** A total of 282 eligible nurses were included in the study with a 100% response rate. Among the respondents, most of 125 44.3%) was in the age range of 25–29 years with mean age of 28.71 [SD ±7.047], more than (half of participants 144 (51.1%) were female, 91 (32.3%) were Amhara by ethnicity, 120 (42.6%) were currently single, 158 (59.6%) had diploma educational status, 157 (58.6%) were orthodox religion members followed by protestant 34.3%. Concerning the respondents working year of experience in the current health institution, most 138 (48.9%) of them had 1–3 years of experience [Table 1].

**Table 1:** Socio-demographic characteristics of participants in public hospitals, South West Ethiopia, 2017/18 (n=282)

Variable	Number	Percent
**Sex**		
Male	138	48.9
Female	144	51.1
**Age in year**		
20-24	73	25.9
25-29	125	44.3
30-34	35	12.4
35-39	25	8.9
>=40	24	8.5
**Marital status**		
Single	120	42.6
Married	106	37.6
Divorced	23	8.2
Widowed	17	6.0
Separate	16	5.7
**Educational status**		
Diploma nurse	168	59.6
Bsc nurse	114	40.4
**Religion**		
Orthodox	165	58.5
Protestant	89	31.6
Muslim	19	6.7
Catholic	9	3.2
**Ethnicity**		
Amhara	91	32.3
Kaffa	53	18.8
Oromo	42	14.9
Sheka	33	11.7
Bench	22	7.8
Tigre	17	6.0
Dawaro	9	3.2
Others *	15	5.3
**Job title**		
Staff Nurse	232	82.3
Specialist Nurse	23	8.2
Head Nurse	27	9.6
**Year of Experience**		
<3	138	48.9
3–5	66	23.4
6–10	56	19.9
11–15	13	4.6
>15	9	3.2

* Gurage, Sidama, Wolayeta

**Organizational and psycho-social related variables**: Among all respondents, more than half of respondents did not get sufficient reward for their work 160 (56.7%), the majority 221 (78.4%) of nurses had good interaction with their co-workers, 237 (88.4%) of them were practiced caring of dying patients, and more than half 150 (53.2%) of the participants were planning to leave their working environment in the near future.

More than half of respondents 155 (55.0%) had an intermediate social support, whereas 65 (23%) and 62 (22%) of them had low and strong social support respectively. From the total participants about 107 (37.9%) had fatigue syndrome, 78 (27.7%) had psychological distress, 244 (86.5%) had problem with perfectionism and 51(18.1%) had major problem with sleep [Table 2].

**Table 2:** Organizational and psycho-social related variables of participants in selected public hospitals, South West Ethiopia, 2017/18

Variable	Frequency	Percent
**Having insufficient reward**		
Yes	160	56.7
No	122	43.3
**Having good interaction with other staffs**		
Yes	221	78.4
No	61	21.6
**Caring of dying patients**		
Yes	248	87.9
No	34	12.1
**Intending to leave working environment**		
Yes	150	53.2
No	132	46.8
**Fatigue**		
No	175	62.1
Yes	107	37.9
**Social support**		
Poor	65	23.0
Intermediate	155	55.0
Strong	62	22.0
**Problem with perfectionism**		
No	38	13.5
Yes	244	86.5
**Psychological distress**		
No	204	72.3
Yes	78	27.7
**Sleeping problem**		
Normal sleep	131	46.5
Minor problems with sleep	100	35.5
Major problems with sleep	51	18.1

**Prevalence of burnout syndrome:** The prevalence of burnout syndrome among nurses was 96(34%) with 95% CI (28%, 40%).

**Factors associated with burnout syndrome:** Based on bivariate analysis variables like; age, educational status, job title, year of experience, intending to leave working environment, fatigue, social support, psychological distress and insomnia were the factors found to be significantly associated with burnout syndrome.

After adjusting for possible confounders with multivariate analysis; educational status, job title, year of experience, fatigue and social support were found to be strong predictor variables for burnout syndrome.

Respondents whose educational status were diploma nurse were 4.8 times more likely to have burnout syndrome as compared with participants with educational status of BSc nurse [AOR= 4.78, 95% CI (2.11, 10.86)]. The odds of having burnout syndrome were almost 15 times more likely in those participants with job title of staff nurse as compared with head nurse [AOR= 14.78, 95% CI (2.46, 8.65)]. Participants whose working experience were 11–15 years were almost 15 times more likely to have burnout syndrome than those participants with experience of 1–3 years [AOR= 14.84, 95% CI (1.01, 79.34)]. Participants with no fatigue were 19.3% times less likely to have burnout syndrome as compared with their counter parts [AOR= .193, 95% CI (.095-.394)]. Besides, participants with poor social support were almost 14 times more likely to have burnout syndrome than those with strong social support [AOR= 13.93, 95% CI (4.07, 47.67)] [Table 3].

**Table 3:** Bivariate and multivariate analysis of predictor variables for burnout syndrome in selected public hospitals, South West Ethiopia, 2017/18

Variables	Burnout syndrome		COR (95%CI)	AOR (95% CI)
	No N (%)	Yes N (%)		
**Age**				
20–24	53(72.6)	20(27.4)	1.00+	1.00+
25–29	83(66.4)	42(33.6)	1.34(.711–2.529)	.908(.404–2.042)
30–34	26(74.3)	9(25.7)	.917(.367–2.293)	.750(.202–2.785)
35–39	11(44.0)	14(56.0)	3.37(1.314–8.655)*	.794(.170–3.709)
>=40	13(54.2)	11(45.8)	2.24(.864–5.819)	.653(.046–9.221)
**Educational status**				
Diploma nurse	90(53.6)	78(46.4)	4.62(2.568–8.319)*	4.78(2.11–10.86) *
Bsc nurse	96(84.2)	18(15.8)	1.00+	1.00+
**Job title**				
Staff Nurse	145(62.5)	87(37.5)	7.50(1.734–32.443)*	14.78(2.46–8.65)*
Specialist Nurse	16(69.6)	7(30.4)	5.47(1.007–29.701)*	2.21(.276–17.628)
Head Nurse	25(92.6)	2(7.4)	1.00+	1.00+
**Year of Experience**				
<3	106(76.8)	32(23.2)	1.00+	1.00+
3–5	45(68.2)	21(31.8)	1.546(.806–2.966)	1.17(.518–2.644)
6–10	25(44.6)	31(55.4)	4.11(2.126–7.937)*	4.663(1.46–4.897)*
11–15	3(23.1)	10(76.9)	11.04(2.864–42.567)*	14.84(1.01–79.34)*
>15	7(77.8)	2(22.2)	.946(.187–4.784)	4.348(.162–66.91)
**Intending to leave** **working environment**				
Yes	91(60.7)	59(39.3)	1.665(1.008–2.749)*	1.879(.882–4.004)
No	95(72.0)	37(28.0)	1.00+	1.00+
**Fatigue**				
No	141(80.6)	34(19.4)	0.175(.102–.299)*	0.193(.095–.394)*
Yes	45(42.1)	62(57.9)	1.00+	1.00+
**Social support**				
Poor	31(47.7)	34(52.3)	8.618(3.417–21.731)*	13.93(4.07–47.67)*
Intermediate	100(64.5)	55(35.5)	4.321(1.842–10.137)*	3.48(1.165–10.41)*
Strong	55(88.7)	7(11.3)	1.00+	1.00+
**Psychological distress**				
No	147(72.1)	57(27.9)	.388(.226–.665)*	.617(.26–1.465)
Yes	39(50.0)	39(50.0)	1.00+	1.00+
**Insomnia**				
Normal sleep	93(71.0)	38(29.0)	.633(.322–.246)*	1.066(.336–3.375)
Minor problems with sleep	62(62.0)	38(38.0)	.950(.475–1.898)	.801(.266–2.413)
Major problems with sleep	31(60.8)	20(39.2)	1.00+	1.00+

* Adjusted for all significant variables at p <0.05

COR= crude odds ratio AOR= adjusted odds ratio, CI = confidence interval

+ Reference Category

## Discussion

This institutional based cross-sectional study was conducted in public hospital to identify the prevalence of burnout syndrome and its associated factors. In addition to this, the findings of this study will have a significant role towards overcoming the problems associated with burnout syndrome among nurses, including decrement of quality of care (26), poor interaction with health care professionals (27), and high turnover rates and absenteeism (28).

In this study, the prevalence of burnout syndrome was 34% and this study was found to be low when it is compared with a study done in Jimma University Teaching Hospital (JUTH), Ethiopia (82.8%)(17), in China (56.03%) (29), and in Spain (69.2%) (30). In contrast to the other study, this study was found to be higher compared with the studies conducted in different areas, where in Italian and Dutch 2% and 10% respectively (19) and in Brazilian 12% (31). The possible explanation for the difference in prevalence may be due to the difference in study setting, study population, tools and methodological differences. For instance, the studies conducted in Brazil and Jimma University Teaching Hospital (JUTH), Ethiopia used Spanish burnout inventory-educational version (SBIEd) and Copenhagen's burnout inventory tool to assess burnout syndrome, respectively, which is different from our tool (MBI-HSS). Moreover, this discrepancy might be due to the fact that Jimma University Teaching Hospital (JUTH) is the hospital which serves for millions of people with the acute and chronic and/or complicated disease as compared with those hospitals where this study was conducted. Therefore, those nurses who are working in Jimma University Teaching Hospital (JUTH) are more prone to develop burnout syndrome due to work overload, and job insecurity. In contrast to this, majority of nurses from developed countries like Italy (19) might be with good educational status, job title and strong social support, whereas, in this study, there were respondents with low social support, fatigue syndrome, psychological distress and major sleeping problem which could possibly contribute for the development of burnout syndrome.

Different independent predictor variables associated with burnout syndrome were identified in this study.

In this study, educational status was found to be significantly associated with burnout syndrome. Respondents whose educational status were diploma nurse were 4.8 times more likely to have burnout syndrome as compared with participants with educational status of Bachelor of Science (BSc) degree nurse. This is congruent with the study conducted in Greece (32) and Amhara, Ethiopia (18). This could be explained as excessive tasks and responsibilities with strict tight schedule were commonly practiced by staffs with low educational level. In contrast to this, having high level of education has been created a good opportunity for nurses to be assigned in a good position where there is satisfactory rewards and other benefits. Therefore, this can possibly be explained as nurses with low educational level are more prone to experience high emotional exhaustion and depersonalization with low personal achievement (33).

Job title was also significantly associated with burnout syndrome. The odds of having burnout syndrome were almost 15 times more likely in those nurses with job title of staff nurse as compared with head nurse. This study finding was consistent with the studies conducted in Ankara, Turkey (34) and in Spain (35). This might be due to the fact that those nurses in the position of staff nurse may experience more burnout and less job satisfaction because they spend more time with patients. Conversely, nurses working in managerial positions, generally have more professional experience and higher level of education, which factors can help them to cope with emotional exhaustion and also increase personal accomplishment and job satisfaction (36).

Working experience was also another significantly associated variable with burnout syndrome. Participants whose working experience were 11–15 years were almost 15 times more likely to have burnout syndrome than those participants with experience of 1–3 years. This finding is in line with the studies conducted in Greece (32), and in Amhara, Ethiopia (18). This might be explained by that nurses who worked for long duration of time in the health setting are more subjected for emotional exhaustion, lack of energy and general fatigue which in turn leads for the development of burnout syndrome (37).

In this study, fatigue was also another significantly associated variable with burnout syndrome. Participants with no fatigue were 19.3% times less likely to have burnout syndrome as compared with their counter parts. This is supported by study from Iran (38). This might be for a reason that nurses with fatigue are commonly experience with devastating sense of drowsiness, lack of energy, and impaired cognitive and/or physical functioning, which may lead to health problem, decrement of performance, increased risk of injury/accident, and low personal achievement, which finally, possibly develop burnout (39).

Finally, social support was also another significantly associated variable with burnout syndrome. Participants with poor social support were almost 14 times more likely to have burnout syndrome than those with strong social support. This finding is also supported by the study conducted in China (29). A plausible explanation to this could be the fact that nurses with good social support can have good mental and physical health (40), where this leads to the decrement of emotional exhaustion and depersonalization with increment of personal accomplishment.

Even though this study contributes as an input for the policy makers towards the decrement of the burnout syndrome among nurses, it has its own limitations. First, since cross-sectional study design was implemented, it can't establish cause effect relationship between the predictor variables and dependent variables. Second, the tools used to assess burnout syndrome was not validated in Ethiopia. Thirdly, this study was not addressed issues related to burnout syndrome qualitatively. Finally, other health professionals were not included, which limited us not to compare nurses with other health professionals. We would like to recommend researchers to do similar studies this time forward considering those limitations.

In conclusion, this study revealed that a considerable proportion of nurses had burnout syndrome. Educational status, job title, year of experience, fatigue and social support were found to be strong predictor variables for burnout syndrome. Therefore, improved educational status and strong social support should be encouraged among nurses working in the health setting for the betterment of health care services, job satisfaction and quality of care in general.
